# Supplementary material for: Application of modular response analysis to medium- to large-size biological systems
Source: PLoS Comput Biol. 2022 Apr 20;18(4):e1009312. doi: 10.1371/journal.pcbi.1009312 (PMC9060349; doi:10.1371/journal.pcbi.1009312)
Supplement: Attachment — Submitted filename: answers-to-reviewers.pdf [file pcbi.1009312.s006.pdf]

Dear Editors,
Dear Reviewers,

Thank you for evaluating our manuscript and for your comments that enabled us to improve our work. Please find hereafter our detailed answers.

During the time of the review, we have found a better implementation of ARACNE that yielded performance worth discussing in the revised manuscript. It has been added in every comparison, figure, and supplementary information accordingly. We also added tests on synthetic data. With two additional figures, we decided to remove the previous figures presenting t-SNE plots of the data sets, which did not play any important role in our study.

Yours faithfully,
Jacques Colinge on behalf of all the authors

Reviewer #1:

The authors have developed a new Modular response analysis method for biological perturbation analysis. The applications to medium and large systems show superior performances to other methods when compared to PPI. The algorithm could also parallel in multi-cores. The paper is well written and easy to follow.

We thank the reviewer for this global positive feedback

Major:

1. In the case study, the authors only claim a difference between tumor and normal cells. The simple difference isn't enough because many methods can produce a difference while the difference isn't not necessarily meanful in biology. This is also a critical point to justify the method.

Thank you for raising this important point. First, we would like to stress that our manuscript is purely methodological and the data sets were selected because they provided systematic perturbations as required by MRA. We provided one example for each data set to illustrate the potential interest of a network biology approach, but our purpose was not to pretend that biologically meaningful conclusions can be derived from these data sets. We simply propose a novel and competitive method to infer biological networks, whose importance in biological research is well-known independent of the two particular data sets exploited in the study to estimate performance solely. The value of the proposed method is justified by comparison with reference data sets of molecular interactions and related, broadly used methods.

Regarding the L1000/CMAP data set, which is the one where we compared a "normal" network to a "metastatic melanoma" network, the particular design of L1000 renders the suggested analysis difficult. Indeed, L1000 profiles the expression of ~1000 landmark genes chosen for their ability to recapitulate whole transcriptomes of the CCLE cancer cell lines. Using the DAVID web server, enrichment analysis of diseases associated with the genes present in the normal HA1E data set returns multiple cancers at the top of the list (GAD database, see below). Accordingly, we did not find obvious cancer signatures in cancer cell lines that would be absent in the (immortalized) normal cell line HA1E with such cancer-biased genes. To return to whole transcriptomes is not possible since MRA requires systematic, individual perturbations of all the genes, what is only available for L1000 hallmark genes.

Functional Annotation Chart

Help and Manual

Current Gene List: genes-HA1E
Current Background: Homo sapiens
937 DAVID IDs
Options

Rerun Using Options | Create Sublist

332 chart records

Download File

Sublist	Category	Term	RT	Genes	Count	%	P-Value	Benjamini
☐	GAD_DISEASE	Breast Cancer	RT		94	10.0	2.0E-20	8.2E-17
☐	GAD_DISEASE	breast cancer	RT		85	9.1	1.1E-19	1.8E-16
☐	GAD_DISEASE	Lung Cancer	RT		92	9.8	1.3E-19	1.8E-16
☐	GAD_DISEASE	Bladder Cancer	RT		85	9.1	4.4E-19	4.5E-16
☐	GAD_DISEASE	lung cancer	RT		82	8.8	4.0E-18	3.3E-15
☐	GAD_DISEASE	esophageal adenocarcinoma	RT		65	6.9	9.0E-18	6.1E-15
☐	GAD_DISEASE	chronic obstructive pulmonary disease	RT		67	7.2	3.9E-15	2.3E-12
☐	GAD_DISEASE	ovarian cancer	RT		62	6.6	9.4E-15	4.8E-12
☐	GAD_DISEASE	plasma HDL cholesterol (HDL-C) levels	RT		49	5.2	1.8E-14	7.5E-12
☐	GAD_DISEASE	Type 2 Diabetes| edema | rosiglitazone	RT		202	21.6	1.8E-14	7.5E-12
☐	GAD_DISEASE	Chronic renal failure|Kidney Failure, Chronic	RT		108	11.5	2.1E-14	7.8E-12
☐	GAD_DISEASE	Colorectal Cancer	RT		64	6.8	5.7E-13	1.9E-10
☐	GAD_DISEASE	Lymphoma, Non-Hodgkin	RT		43	4.6	7.5E-11	2.4E-8
☐	GAD_DISEASE	Alzheimer's disease	RT		101	10.8	1.8E-10	4.9E-8
☐	GAD_DISEASE	Multiple Sclerosis	RT		72	7.7	1.8E-10	4.9E-8
☐	GAD_DISEASE	epithelial ovarian cancer	RT		34	3.6	2.0E-10	5.0E-8
☐	GAD_DISEASE	ovarian cancer	RT		31	3.3	2.6E-10	6.3E-8
☐	GAD_DISEASE	Colorectal Neoplasms	RT		24	2.6	7.0E-9	1.6E-6
☐	GAD_DISEASE	null	RT		94	10.0	1.6E-8	3.4E-6
☐	GAD_DISEASE	Leukemia, Lymphocytic, Chronic, B-Cell	RT		35	3.7	1.9E-8	3.8E-6
☐	GAD_DISEASE	HIV	RT		34	3.6	3.9E-8	7.6E-6
☐	GAD_DISEASE	Bone Mineral Density	RT		51	5.4	1.2E-7	2.2E-5
☐	GAD_DISEASE	longevity	RT		52	5.5	1.4E-7	2.4E-5
☐	GAD_DISEASE	Arthritis, Rheumatoid|	RT		26	2.8	1.5E-7	2.6E-5
☐	GAD_DISEASE	atherosclerosis	RT		44	4.7	1.0E-6	1.7E-4
☐	GAD_DISEASE	diabetes, type 2	RT		50	5.3	2.1E-6	3.2E-4
☐	GAD_DISEASE	Head and Neck Neoplasms|Neoplasm Recurrence, Local|Neoplasms, Second Primary	RT		24	2.6	2.1E-6	3.2E-4
☐	GAD_DISEASE	Alzheimer's Disease	RT		41	4.4	3.0E-6	4.4E-4
☐	GAD_DISEASE	Precursor Cell Lymphoblastic Leukemia-Lymphoma	RT		21	2.2	3.4E-6	4.8E-4
☐	GAD_DISEASE	stomach cancer	RT		19	2.0	4.5E-6	6.1E-4
☐	GAD_DISEASE	Brain Neoplasms|Glioma|meningioma|Neuroma, Acoustic|Neuromas, Acoustic	RT		7	0.7	4.9E-6	6.4E-4
☐	GAD_DISEASE	Inflammation|Premature Birth	RT		22	2.3	6.8E-6	8.3E-4
☐	GAD_DISEASE	Connective Tissue Diseases|Fetal Diseases|Inflammation|Musculoskeletal Diseases|Pregnancy Complications, Hematologic|Premature Birth|Skin Diseases	RT		22	2.3	6.8E-6	8.3E-4
☐	GAD_DISEASE	Esophageal Cancer	RT		14	1.5	1.0E-5	1.2E-3
☐	GAD_DISEASE	Stomach Neoplasms	RT		20	2.1	1.3E-5	1.5E-3
☐	GAD_DISEASE	prostate cancer	RT		52	5.5	2.8E-5	3.2E-3
☐	GAD_DISEASE	Mouth Neoplasms|Precancerous Conditions	RT		8	0.9	2.9E-5	3.2E-3
☐	GAD_DISEASE	head and neck cancer	RT		17	1.8	3.5E-5	3.7E-3
☐	GAD_DISEASE	metabolic syndrome	RT		24	2.6	3.9E-5	3.9E-3
☐	GAD_DISEASE	Brain Neoplasms|Glioblastoma	RT		9	1.0	3.9E-5	3.9E-3
☐	GAD_DISEASE	Albuminuria|Inflammation|Kidney Diseases	RT		9	1.0	3.9E-5	3.9E-3
☐	GAD_DISEASE	Hodgkin Disease|Leukemia, Lymphocytic, Chronic, B-Cell|Lymphoproliferative Disorders|Waldenstrom Macroglobulinemia	RT		21	2.2	4.1E-5	4.0E-3
☐	GAD_DISEASE	Carcinoma, Squamous Cell|Mouth Neoplasms|Neoplasm Metastasis|Squamous cell carcinoma	RT		5	0.5	4.5E-5	4.2E-3
☐	GAD_DISEASE	Type 2 diabetes	RT		40	4.3	4.5E-5	4.2E-3
☐	GAD_DISEASE	overall effect	RT		8	0.9	6.4E-5	5.6E-3
☐	GAD_DISEASE	pancreatic neoplasm|Pancreatic Neoplasms	RT		8	0.9	6.4E-5	5.6E-3
☐	GAD_DISEASE	myeloid leukemia	RT		9	1.0	7.3E-5	6.3E-3

2. The method was evaluated with different proportions of predicted interactions. While the higher accuracy is valuable, it's required to describe the evaluate the obtained values like p-values. Such values can provide a guide threshold for users to use the results.

We certainly agree, but there are different factors to consider. First of all, the different algorithms we compared do not estimate the confidence of individual inferred interactions. For this reason and to homogenize the comparisons, we decided to take the top 5%, 10%, 20%, etc. scored interactions of each algorithm and compare to a reference database. These comparisons yield confusion matrices in every case, for which we computed a P-value reflecting the overall quality of the inference at a top x% level for each algorithm. All the confusion matrices were provided. The revised text insists on this point to make sure it is clear for the reader thanks to this remark. (We also provide additional indicators sur as specificity, precision, etc.)

Second, to better address this important point, we have introduced synthetic networks as an initial estimate of performance. Indeed, there is a standard difficulty dealing with experimental data, which is that the reference network is not known accurately and completely. Moreover, such networks depend on the very genome of the cells used (especially in the L1000/CMAP data set with distinct cancer cell lines), and their culture conditions (especially the medium size kinase data set). Reference public interaction databases aggregate observations made in a few convenient cell lines such as HEK293, etc. This was the reason to use STRING due to its ability to

be more “comprehensive” than other resources. Nevertheless, missing and false positive interactions in STRING renders the estimation of accurate TP, FP, TN, and FN interactions impossible. Only approximations can be obtained. We believe that this is not an issue since all the methods are compared with the same procedure (as many other authors do in identical conditions).

We believe that the complement provided by performance estimates on synthetic networks combined by the summary figures and all the confusion matrices (with P-values, precision, etc.) should be sufficient to support our claims and let readers make their opinion.

3. It’s beneficial to show the actual running time and make comparison with other methods.

We have added a table and discussed this information.

Reviewer #2:

This paper proposed to use MRA to infer the protein interaction network using the data obtained through the33 systematic perturbation of the actors. While the topic is interesting, I have a few concerns as listed below.

1. In fig 1, the green cluster seems to corresponding to FGF1, instead of WNT3A?

The text indeed contained a few mistakes referring to these colors; we apologize for this. This part has been removed from the revised version since it was not really useful and we introduced two new figures.

2. For the data there are 61genes and 11 conditions. In my understanding, there should be 11 networks estimated. Or have you inferred the networks for None-, WNT3A- and IFNg-stimulated data separately?

We inferred separate networks for each condition, *i.e.*, 11 networks. Those inferences were done independently since we do not know how much is shared (kinase signaling might be pretty flexible) and, anyway, we only propose an algorithm to infer one network from one dataset. We do have a notion of increased confidence on shared features between parallel inferred networks that might have similarities. This would be a nice feature but it is out of our scope. We are sorry if the text was not completely clear; we have revised it to better insist on this important detail.

3. For STRING database, some of their interactions are predicted with a confidence score. Did you use any threshold in the STRING network? If so, how did you select the threshold?

This is an important point, which we have addressed. In the revised manuscript, all the comparisons against STRING that are reported in the figures, correspond to STRING interactions with STRING score > 0.5, a reasonable compromise between stringency and sensitivity of the reference network. In the supplementary tables, we report all the confusion matrices against STRING with scores > 0.8, 0.5, and 0. This does not change the relative merit of each algorithm and is now mentioned in the revised text.

4. For the performance evaluation, it is better to include other metrics like specificity, sensitivity, AUC and accuracy as well.Without these information, it is hard for me to evaluate the quality of this work properly.

We agree and the tables in the figures have been modified to include specificity, accuracy and precision. All the supplementary tables have been updated as well, they contain in addition recall and P-values.

Have the authors made all data and (if applicable) computational code underlying the findings in their manuscript fully available?
Reviewer #1: None
We made the code available with one example data set (ACTA condition from K61 data set). We have now added the 50TFx50TA synthetic data example.

Reviewer #2: Yes
